# Protocol for a randomized trial to predict the efficacy of cognitive and behavioral interventions for symptoms of depression

**DOI:** 10.3389/fpsyt.2026.1774560

**Published:** 2026-04-16

**Authors:** Jialing Ding, Jamie C. Chiu, Seohyun Moon, Yongjing Ren, David M. Turner, Gal Shoval, Yael Niv, Isabel M. Berwian

**Affiliations:** 1 Princeton Neuroscience Institute, Princeton University, Princeton, NJ, United States; 2 Department of Psychology, Princeton University, Princeton, NJ, United States; 3 Child and Adolescent Clinic, Geha Mental Health Center, Petah Tikva, Israel; 4 Gray Faculty of Medical & Health Sciences, Tel Aviv University, Tel Aviv, Israel; 5 Research Department of Clinical, Educational and Health Psychology, University College London, London, United Kingdom

**Keywords:** cognitive behavioral therapy (CBT), depression, prediction model, psychotherapy, randomized clinical trial

## Abstract

**Introduction:**

Cognitive behavioral therapy (CBT) is one of the most common interventions for depression and has two key components: Cognitive Restructuring (CR) and Behavioral Activation (BA). However, no evidence-based guidelines exist to help clients and clinicians decide whether CBT would be a good first-line treatment for a given individual based on their personal characteristics, and which CBT intervention would benefit them more. We propose that specific capacities to learn from new information and experiences are prerequisites for response to CBT and that BA and CR require different learning capacities. In this study, we aim to develop predictive models of symptom change based on computationally-derived variables from behavioral tasks, in addition to clinical and demographic self-report data, to identify parameters and variables that can determine which individuals with depressive symptoms would benefit from CBT-based interventions and, ideally, which specific interventions they would benefit from more.

**Methods and analysis:**

We plan to recruit at least 1,500 adult participants who report having symptoms of depression and reside in U.S. After completing a series of questionnaires and behavioral tasks to assess their learning propensities, participants will be randomly assigned to a BA or a CR group. Using an online self-help tool, participants will then engage with designated modules according to their assigned group for five weeks. We will assess symptoms 1 week post-intervention (main end point of study) and follow up at 6, 18, and 42 weeks post-intervention. Upon enrolling and consenting into the main study, participants will be randomly assigned to either the training dataset or the held-out test dataset at a ratio of 2:1. This enables a clean separation of training and test datasets and prevent data leakage. We plan to build cross-validated predictive algorithms on the training dataset, and preregister our analysis plan before we validate our models and hypotheses in the held-out, unseen, test dataset. Enrollment of the study started 23rd January, 2024.

**Study protocol registration:**

ClinicalTrials.gov, identifier (NCT06631183). The protocol follows the Standard Protocol Items: Recommendations for Interventional Trials (SPIRIT) guidelines. Numbers in brackets follow subsection numbers in the guidelines.

## Introduction

### Background and rationale (9a)

Depression is a prevalent and debilitating disorder associated with considerable morbidity, mortality, and socioeconomic costs (1). Alongside antidepressants, psychotherapy is an effective and established treatment for depression (2, 3). Cognitive behavioral therapy (CBT)—an umbrella term encompassing interventions developed across different theoretical “waves,” including cognitive interventions and behavioral activation (4–6)—is among the most widely applied therapeutic approaches for depression.

Current treatment guidelines reflect this prominence. For instance, the National Institute for Health and Care Excellence (NICE) (7) recommends a range of possible first-line treatments for less severe depression, most of which are CBT-based interventions. However, it provides no specific criteria for selecting between these options beyond “clinical needs and preferences, taking into account that any option can be used as first line, but consider the least intrusive and least resource intensive treatment first (guided self-help)” (7, Section 1.4.2). Indeed, it is unclear which of several cognitive or behavioral interventions an individual is most likely to benefit from. Consistent with well-powered trials showing comparable efficacy across psychotherapy approaches (8, 9) and therapy components (10, 11), the 2009 NICE guidelines similarly acknowledged that “there is little evidence to guide prescribing in relation to depression subtypes or personal characteristics” (12, Section 1.6).

Up to half of patients who receive CBT show only partial or minimal improvement (13). As a result, many individuals undergo lengthy treatment without meaningful benefit, some of whom might have improved more with a different therapeutic approach. Importantly, such average response rates can mask substantial individual variability. Two interventions may have similar mean effectiveness – for example, both CBT and interpersonal therapy (IPT) are similarly effective, each improving symptoms in around 50% of patients (14, 15) – yet the subsets of individuals who benefit from each may differ markedly (e.g. see 16, 17). One intervention might work particularly well for a specific subgroup, while another benefits a different subgroup; some individuals might respond to both, and others to neither.

If it were possible to identify *a priori* which intervention is best suited for which individual, the overall response rates could rise substantially, by matching each person to the intervention most aligned with their characteristics (18, 19). Improving the ability to predict who is likely to respond to which intervention could therefore enhance treatment outcomes and make more efficient use of clinical resources. Accordingly, there is growing interest in and urgent need for developing personalized treatment recommendations in psychotherapy.

Initial evidence suggests that certain self-report measures (20, 21) and neural activity patterns (22, 23) may be associated with response to CBT. However, self-report predictors can be biased and often show low reliability in meta-analyses (e.g., 24). From a neurobiological perspective, neuroimaging markers have not been shown to meaningfully improve predictive accuracy when added to clinical variables (especially when accounting for small sample sizes) (25). Moreover, neuroimaging is unlikely to be practical in the clinical setting given its cost and complexity relative to the potential gains in prediction.

Finally, we note that previous work has shown that self-report measures are more related to each other than to task-derived data (26). That is why it is important to include such measures in all our models as a baseline. However, in our own prior work, we showed that while standard clinical variables (many of which were self-report or based on self-reported information) did not predict subsequent episodes of depression (27), behavior derived from an Effort Task (included in this study) improved predictive accuracy out-of-sample, and in data from a second site (28).

### CBT as a learning-based framework with distinct mechanisms

CBT can be conceptualized as a learning-based therapeutic framework that lends itself to examination through computational models of learning (see 29, 30). CBT aims to trigger learning of new behavioral, cognitive and emotional responses. Successful CBT treatment therefore depends on intact learning mechanisms and neural flexibility within the circuits the treatments aim to modify.

Within this framework, different CBT components engage distinct forms of learning, providing an opportunity to examine how specific therapeutic techniques operate through separable underlying mechanisms. Among these, Behavioral Activation (BA; 4, 31) and Cognitive Restructuring (CR; 4, 5) exemplify two distinct routes for therapeutic learning to help alleviate symptoms of depression.

A major aim of BA is to increase engagement in rewarding activities to counteract avoidance and anhedonia through experiencing the benefits of such engagement, whereas CR targets maladaptive thought patterns by helping individuals identify and modify negative interpretations and beliefs. Both interventions are effective as stand-alone treatments (32, 33), yet they rely on distinct underlying learning and neurocognitive mechanisms.

We assume that in BA, therapeutic change occurs primarily through experience-based reinforcement learning. The intervention seeks to correct distortions in the anticipation of reward and effort by encouraging patients to re-engage with potentially pleasurable, but effortful, activities. Through repeated trial-and-error experiences, individuals update the anticipated costs and benefits associated with their actions – an example of model-free learning driven by dopamine-mediated prediction error signaling (33). BA may therefore be most effective for those who incorrectly anticipate high effort and low reward for activities that they will find enjoyable and not too difficult to execute (34).

In contrast, CR may rely primarily on deliberative, cognitive learning processes. CR helps individuals recognize automatic negative thoughts, challenge them using counter-evidence, and re-evaluate maladaptive beliefs about themselves and their environment. This process aligns with model-based learning, which depends on the hippocampus and prefrontal cortex and is largely dopamine-independent (30). However, changing explicit beliefs alone may not automatically translate into new emotional or behavioral responses, which are often governed by implicit action values. Thus, individual differences in the ability to translate cognitive insight into implicit behavioral change may influence the effectiveness of CR (29).

Accordingly, our study is based on the hypothesis that individual differences in learning mechanisms, including reinforcement-based and cognition-based learning, and related cost/benefit decision variables, can predict which individuals would benefit most from CBT-based interventions and which subtype of CBT would be most beneficial for a given individual. Identifying such moderators could enable mechanism-based, differential intervention selection.

### Explanation for choice of comparator (9b)

The goal of our study is not to test whether BA or CR is superior overall, but rather to identify which individuals benefit more from each approach. BA and CR were chosen because they represent core, manualized components of CBT for depression. Although both operate within the same therapeutic framework, they are theoretically dissociable in terms of learning mechanisms (i.e., reinforcement-based vs. cognitive-based). These interventions are also thought to engage distinct brain regions and cognitive processes that underpin the learning mechanisms they trigger (29, 30, 34). Comparing these two interventions thus allows examination of differential predictors of treatment response while holding the overall therapeutic framework constant.

### Objectives (10)

The goal of our study is to test whether individuals’ learning propensities and cost/benefit decision variables can predict whether they will respond to CBT-based interventions and which CBT component, BA or CR, is more beneficial for the individual. We broadly hypothesize that:

1. Flexibility and intact learning mechanisms predict better response to CBT, as a learning-based intervention.
2. Reduced anticipation (but intact experience) of rewards and/or increased anticipation (but intact experience) of effort predict better response to BA.
3. The effectiveness of CR depends on the degree to which changes in explicit beliefs translate into changes in implicit affective and behavioral responses.

To assess the learning abilities that we hypothesize to be related to CBT, and to BA and CR specifically, we will employ a set of behavioral tasks in individuals with symptoms of depression and apply computational models to infer their underlying learning and decision-making mechanisms at baseline, prior to engagement with any intervention. Participants will then be randomized in a 1:1 ratio to engage with modules focusing on BA or CR on the self-help platform e-couch for five weeks. We will measure symptoms of depression and associated symptoms before the intervention and after six weeks, which we consider the end point of the main study (although we plan symptom follow-ups up to week 48, see below).

No prior evidence exists that links our main measures of interest, i.e., computationally-derived learning and decision-making parameters from a set of behavioral tasks, to outcomes of BA and CR, and even less so on how these measures interact with covariates such as clinical symptoms and history to predict differential out-of-sample outcome of the interventions. Hence, we will randomize participants to a training and test dataset in a ratio of 2:1 at the beginning of the main study, build cross-validated predictive algorithms on the training dataset and then preregister our precise hypotheses (embedded in the algorithm code) prior to accessing and applying them to the test dataset.

Our planned trial will employ an online, self-guided self-help tool (e-couch) to deliver the interventions. This format allows us to control the intervention delivery and ensure that observed effects are attributable to individual differences in response to the assigned intervention, rather than differences in the delivery of the intervention among participants. The ultimate goal of our work is to contribute to the development of generalizable, out-of-sample predictive algorithms that can determine, for a new individual, whether they would benefit from CBT-based interventions and which intervention (BA or CR) is more likely to produce a benefit for them. This work aims to establish a foundation for precision mental health, enabling personalized, mechanism-based psychotherapy recommendations. Our present trial is the first step in that direction, where we aim to assess and evaluate whether our variables and models have predictive potential. If we succeed with this step, independent clinical trials and implementation studies are needed to make such personalized recommendations a reality.

## Methods: patient and public involvement, trial design

### Patient and public involvement (11)

We recruited two individuals with lived experience of depression to serve as advisors for the trial. Since this practice is still relatively uncommon in the U.S., we handled recruitment by advertising at a study clinic that is not used for participant recruitment. Potential advisors were interviewed with a focus on their ability to reflect on their experiences as patients with depression, as well as their interest in contributing to research aimed at improving treatment. Once selected, the advisors received access to all study materials, including questionnaires, behavioral tasks, the online self-help tool, and the content of emails to be sent to participant in the same sequence that study participants would be exposed to these. The advisors were asked to provide detailed feedback on the design of the tasks, the overall workload, and the clarity of the instructions and language used throughout the study. Importantly, they offered input from their perspective as individuals with lived experience of depression, focusing on whether the materials were emotionally sensitive, inclusive, and accessible. Several aspects of the study materials were adjusted in response to this feedback. Advisors were paid for their time, and will be acknowledged in reporting of results.

### Trial design (12)

This is a randomized trial with two parallel intervention arms. Participants will be randomly assigned to either the Behavioral Activation (BA) group or the Cognitive Restructuring (CR) group in a 1:1 ratio. The primary goal of the study is to determine (1) which baseline characteristics predict the benefits (in terms of reduction of depression symptoms) each individual participant will receive from CBT-based interventions, and (2) which baseline characteristics moderate the therapeutic effects of BA and CR. Therefore, the study does not fall in the categorization of superiority, non-inferiority, or equivalence framework, and we do not make any assumptions regarding differences in overall treatment efficacy. We do not plan to establish efficacy, safety of feasibility. Instead, this is a mechanistic clinical trial aiming to uncover moderation of intervention effects.

## Methods: participants, interventions, and outcomes

### Trial setting (13)

The study will be conducted from Princeton University, NJ. However, all data collection and participant contact will be held entirely online. Participants from the general population from the US will be recruited via online recruitment methods (see section 20, *Recruitment*).

### Eligibility criteria (14a)

Participants are eligible to participate only if they meet all the following criteria assessed by a screening survey at the beginning the study (see **Supplement *Study-specific measures***):

- at least 18 years old,
- physically located in the US,
- fluent in English,
- no hearing problems,
- have access to headphones,
- have not previously participated in studies from our lab,
- no one from the same household has enrolled in this current study,
- are not currently receiving psychotherapy or have plans to begin within the next eight weeks,
- have not changed the dosage or type of antidepressants (if they are on any) in the last four weeks, or plan to make any such changes during the next eight weeks, and
- their primary mental health concern is to improve their mood, reduce negative thoughts, enjoy more activities again, or reduce symptoms of depression.

The above list has been updated from protocol version 1.0 after we examined the data quality from the first 100 participants (See **Supplement *Protocol changes*** for details). Our primary target population will consist of participants who self-report depressive symptoms, defined as a score greater than 4 on the Patient Health Questionnaire (PHQ-9; 35). We will oversample participants meeting this criterion, such that approximately 95% of the sample will fall within this range. Participants with a lower PHQ-9 score will be recruited for practical reasons (namely: as we do not want to encourage over-reporting of symptoms, we explicitly instruct participants that symptoms are not a prerequisite for the study; as such, we must admit to the study some participants without symptoms of depression). However, participants with a PHQ-9 score below 5 are not part of the intention-to-treat sample and will not be analyzed in primary prediction analyses (see section 27b, *Who will be included in the analyses*). To further ensure data quality, we include an attention-check question embedded within the PHQ-9 (see Section 25 *Data collection method*, for an example) and will not include participants who fail the attention check.

### Exclusion after starting the study

Once participants have enrolled in the study, we will exclude those who fail the zoom identity check, demonstrate inattentive task performance, or show insufficient study engagement (see Section 25, *Data collection method: Processes to promote quality of data collection*, for details).

### Intervention and comparator (15a)

Participants will complete five weeks of either the BA or CR portion of the Depression Program on e-couch (http://www.ecouch.com.au), an online, self-guided platform offering interactive modules and evidence-based information to help users understand and manage common mental health symptoms. The Depression Program includes a comprehensive psychoeducation component and multiple self-help modules with interactive exercises and workbooks that teach evidence-based strategies to manage depressed mood (see below for details).

In Week 1, all participants will complete the *Depression Information* module, which provides general, evidence-based psychoeducational information about depression and its symptoms, serving as a foundation for the intervention. From Week 2 onward, participants will access only the modules corresponding to their assigned group. Each prescribed section is expected to take approximately up to 30–60 minutes per week to complete.

The BA group will complete the *Changing Your Behavior* submodule of the *Cognitive Behavioral Therapy* module in week 2, where they will learn to identify things they enjoy and make a schedule of enjoyable activities. They will repeat the same submodule in week 3 so that they will gain a deeper understanding of the strategies. In week 4, the BA group will complete the *Physical Activity* module, where they will evaluate their own level of activity and explore strategies for increasing or maintaining their activity. In week 5, they will be asked to continue with the same module and given the choice to proceed to the next level of the module (i.e., to increase their physical activity), repeat last week’s level, or reduce the level if the previous week’s plans were too ambitious.

The CR group will complete the *Thinking About Thinking* submodule of the *Cognitive Behavioral Therapy* module in week 2, where they will learn to identify their negative thinking and learn strategies to tackle negative thinking. They will repeat this submodule in week 3. In week 4, they will complete the *Changing Your Thinking* submodule of the *Cognitive Behavioral Therapy* module, where they will learn to challenge their negative thoughts and assumptions, and to reframe thoughts to be more realistic or constructive. They will repeat the same submodule in week 5 to review the material, revisit homework and workbook exercises, and deepen their understanding of the concepts. Participants will be encouraged to actively engage with the material and exercises by reflecting on situations they encountered in the previous week.

Previous research has demonstrated that e-couch is effective in reducing depressive symptoms, with meaningful within-subject effect sizes and modest-to-small between-group effects relative to control conditions (36–38). Notably, the BA and CR modules in e-couch were designed to reflect the core therapeutic strategies and mechanisms of change that underpin traditional face-to-face CBT. Following manuals of traditional CBT, both groups first learn about depression and treatment options in the psychoeducation module. In the CR interventions, participants then learn about biased ways of thinking that often occur in depression, how they can identify these, and how they tackle and change these biases. In the BA interventions, they learn about the role of rewarding activities in relation to mood, how to schedule and execute pleasant activities, and later, how they can incorporate more effortful and physical activities that will have a positive impact in the long run. The choice and order of modules was determined by two trained therapists on the study team (IMB, JCC) and in discussion with collaborators who are also trained therapists. Through testing and a pilot study, we ensured that both arms require approximately the same amount of engagement and thus both interventions have a comparable dose. One advantage of delivering these components in a fully automated online format is that every participant receives the same standardized content, which enhances the fidelity of the intervention. Automation also minimizes the unintentional blending of strategies that can occur in therapist-delivered psychotherapy, where individual therapists may naturally shift between techniques or emphasize some elements over others. We will ask participants to repeat the core modules or submodules to ensure they thoroughly practice the skills they learn and reflect on them after trying them out in real life, mimicking procedures in therapist-delivered CBT.

After the five-week intervention period, and once symptoms have been reassessed, participants will maintain their access to e-couch for another five months and will be told they are free to explore any of the remaining modules within the e-couch Depression Program (i.e., *Interpersonal Psychotherapy*, *Problem-solving*, *Relaxation*, and *Workbook*), including those completed by the group to which they were not assigned. They may also revisit and repeat any modules they have already completed. Throughout the study, participants will be allowed to engage in any usual care or treatment they are already receiving, in addition to the assigned e-couch interventions. Changes in usual care during the study will be assessed after the end of the intervention, and in follow-up assessments.

### Criteria for discontinuing or modifying allocated intervention (15b)

We do not plan to alter the allocated interventions based on participants’ responses to the intervention. However, we will encourage participants to engage with other interventions (outside the study) if they find that necessary. Participants may request a break or delay in their participation of up to four weeks at any point during the study.

### Strategies to improve adherence to intervention (15c)

Participants will receive automated reminder emails to encourage timely completion of tasks and questionnaires. Participants will have one week to complete each component with an automatic extension of up to one more week. As such, they will receive up to four reminder emails in total after the initial invitation to complete a part of the study, one reminder approximately every 3 to 4 days. If participants do not respond within 48 hours after the final reminder (sent on day 14), they will be automatically excluded from the study, but will still be compensated for time spent on the study so far.

For the e-couch modules, an automated reminder will be sent three days after the initial invitation. Since e-couch progress is managed by an external platform, we will not track participants’ activity on e-couch in real time. Therefore, each new email will remind participants to complete any outstanding modules from the previous week and encourage them to contact us if an extension is needed. To ensure participants access the correct modules, each email will include clear instructions and visual guides as to what to complete on that week. We will remind participants that following these instructions carefully is required to earn the $10 bonus. We will also instruct them to reach out to the research team if they encounter any issues or realize they have not followed the instructions correctly.

We will assess participants’ adherence to their assigned intervention condition after data collection is complete. Adherence will be defined based on the amount of engagement with the assigned interventions (time spent in e-couch modules and number of logins into the e-couch platform) and deviations from the assigned intervention protocol.

### Concomitant care that is permitted or prohibited during the trial (15d)

Participants may continue any other care they were receiving or engage in additional care, that is, “usual care” will be permitted in both groups. At the end of the main study (week 6) and during follow-up visits, we will ask participants to report any care they received using the Psychiatric Treatment Questionnaire (see **Supplement *Study-specific measures***).

### Outcomes (16)

Our primary outcome is NOT a change in a specific symptom score or the superiority of one intervention over another. Instead, we aim to predict end-of-main-study (week 6) depression symptoms – both overall, and differentially for the BA and CR groups – in a held-out test dataset. Thus, our primary outcome is the out-of-sample predictive accuracy of improvement of depression symptoms using potential moderators. The prediction models will incorporate self-reported questionnaire data (clinical and demographic information) and computationally derived learning and decision-making variables from the behavioral tasks. Thus, our specific outcome depends on the exact target question and is tightly intertwined with the employed analysis approach. For more details on the specifications of the analysis approach and outcomes, see Section 27, *Statistical methods*. Also note that our primary outcome, as well as all trial estimands, will be precisely defined in the preregistration of the analyses of our untouched test dataset (and corresponding code). The separation of our data into a training and test dataset from the beginning of the study makes the specific definition of primary and secondary outcomes redundant at this stage.

### Harms (17)

The study will be conducted entirely online. Thus, we will only be informed about any possible harm that occurred if participants contact us via email or phone. Hence, harms will be assessed unsystematically. The PI, who is blinded to the allocation group, will decide whether the reported harm exceeds the threshold to be considered an adverse event. In such cases, we will report the event to the IRB and follow their guidelines regarding the adverse event.

### Participant Timeline (18)

**Figure 1:**
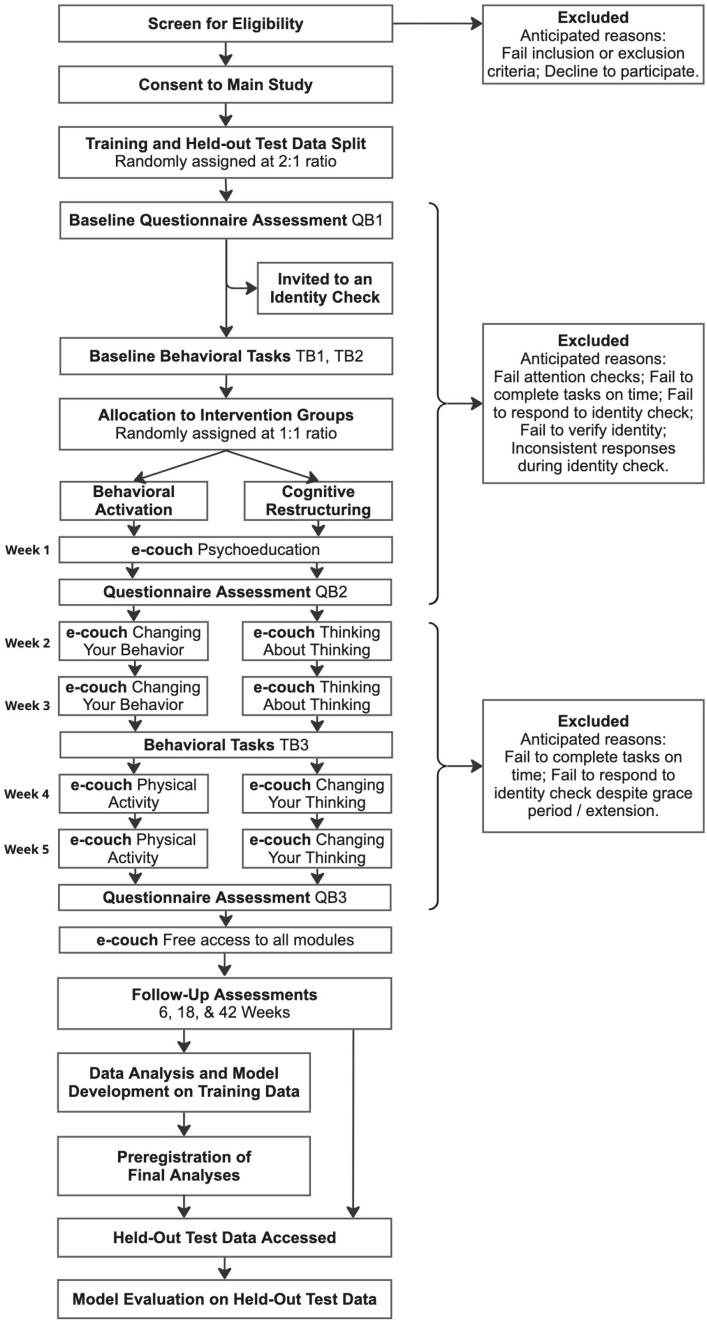
Schematic diagram of study design and participant timeline.

Participants who meet eligibility criteria after the screening survey will be invited to join the main study. After consenting to the main study, they will immediately be randomly assigned to either the training or the test group. The main study will last six weeks (more if extensions are requested), including three questionnaire batches (QB1, QB2, QB3), three task batches (TB1, TB2, TB3), and five weeks of engagement with the e-couch program (see **Figure 1** for the study design and timeline). We use the term *batch* to refer to a set of questionnaires and/or tasks administered at the same timepoint. Each batch should be completed within seven days. An additional one-week extension will be automatically allowed for the completion of each batch, and longer extensions (up to one month) may be granted upon request.

At baseline, participants will complete QB1 (clinical symptom measures and a matrix reasoning task; approximately 35 minutes). They will then proceed to TB1 (four behavioral tasks; approximately 65 minutes) and after one day, to TB2 (two behavioral tasks; approximately 40 minutes). After completing TB2, participants will be randomly assigned to either the BA or CR group in a 1:1 ratio and can begin their five-week engagement with the e-couch program. Each module or submodule will require approximately up to 30–60 minutes per week, and participants will be encouraged to practice skills for a full week before progressing to the next module (see Section 15a, *Intervention and Comparator* for e-couch schedule).

While working through e-couch, participants will also complete additional assessments and behavioral tasks. Before starting e-couch week 2, they will complete QB2 (clinical symptom measures; approximately 7 minutes). Before e-couch week 4, they will complete TB3 (same questionnaires as in QB2 and four behavioral tasks; approximately 70 minutes). At the end of five weeks of e-couch, participants will complete QB3 (clinical symptom measures; approximately 30 minutes) to assess post-intervention symptoms.

**Figure 2:**
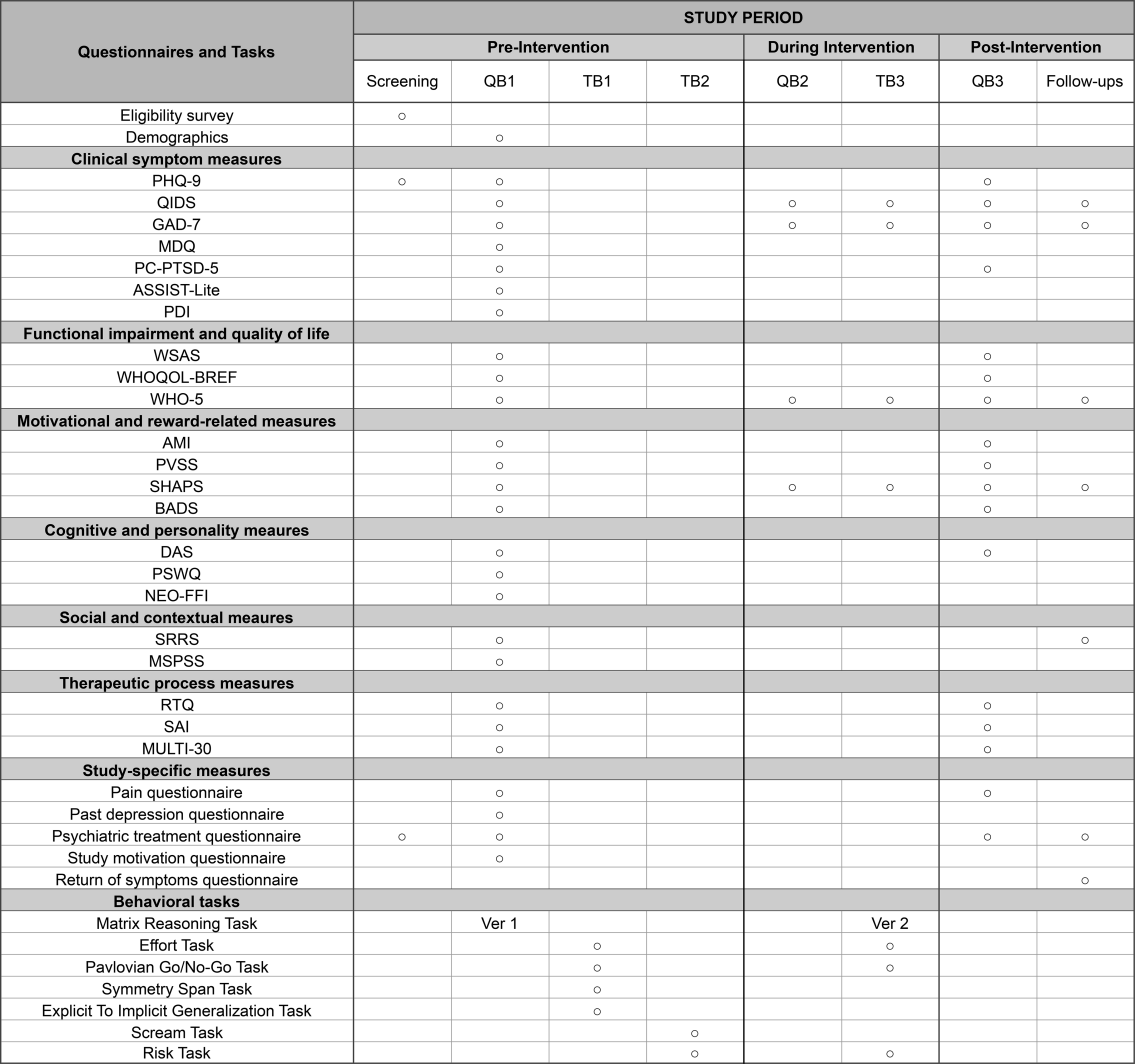
Questionnaires and tasks administered at each study phase. PHQ-9, Patient Health Questionnaire; QIDS, Quick Inventory of Depressive Symptomatology; GAD-7, Generalized Anxiety Disorder 7-item Scale; MDQ, Mood Disorder Questionnaire; PC-PTSD-5, Primary Care PTSD Screen; ASSIST-Lite, Alcohol, Smoking and Substance Involvement Screening Test; PDI, Peters Delusion Inventory; WSAS, Work and Social Adjustment Scale; WHOQOL-BREF, World Health Organization Quality of Life-BREF; WHO-5, World Health Organization-Five Wellbeing Index; AMI, Apathy-Motivation Index; PVSS, Positive Valence Systems Scale; SHAPS, Snaith-Hamilton Pleasure Scale; BADS, Behavioral Activation for Depression Scale; DAS, Dysfunctional Attitude Scale; PSWQ, Penn State Worry Questionnaire; NEO-FFI, NEO Five Factor Inventory (Neuroticism subscale); SRRS, Social Readjustment Rating Scale; MSPSS, Multidimensional Scale of Perceived Social Support; RTQ, Readiness for Therapy Questionnaire; SAI, Session Alliance Inventory; MULTI-30, Multitheoretical List of Therapeutic Interventions. References for all questionnaires and tasks are provided in section 25, *Data collection method*.

Follow-up of symptoms will be assessed with questionnaires at 6, 18, and 42 weeks after study completion to assess immediate, mid-term, and long-term effects of the e-couch intervention. Each questionnaire will take approximately 7 minutes. For detailed description of the questionnaires and behavioral tasks, see **Figure 2** and Section 25, *Data collection method*.

### Sample size (19)

We computed the required sample size for a multivariable prediction model with binary outcomes based on Riley et al. (39). To our knowledge, previous models are not available in the literature to anticipate Cox-Snell R2 for our study. Hence, we estimated the required Cox-Snell R2 value to be 0.05 based on pseudo-R2 values from the field of computational psychiatry, assuming small effect sizes when relating task behavior to symptom measurements (40, 41). Note, however, that this is a very conservative estimate, as we have previously found a large effect size when relating parameter estimates from the planned effort task to clinical diagnoses and a medium effect size when relating the parameters to subsequent symptom development (28). Furthermore, as we have optimized our tasks to relate to symptom changes due to therapy, we expect larger effect sizes in this study. For the sample size calculation, we also assumed a prevalence of response to iCBT of 0.5 (42), setting the shrinkage parameter to 0.9 to avoid overfitting 4–6 predictors (parameter estimates from our tasks and candidate predictors from the literature such as baseline severity (43) and gender (42)). The computed required sample size is 875 participants. We have therefore conservatively planned for a training set sample size of N = 1000. For current planning purposes, we assume that we will dedicate data from over 300 participants to the test dataset, well exceeding the recommendations for 100–200 events for validation data sets (44). Anticipating an attrition rate of approximately 20% (45, 46), we aim to recruit a total of 1,500 participants based on power analysis. We will compute the power that our test dataset provides once we have obtained results from our model development (47).

### Recruitment (20)

We will advertise the study on ResearchMatch, popular social media platforms (Instagram, Facebook, X, Reddit, and TikTok), the newspaper (USA Today), and research marketplace (through Cint). Recruitment will take place entirely online. Interested individuals will be able to visit the study information page on our lab website, where they will find a link to the screening survey.

ResearchMatch is a national health volunteer registry that was created by several academic institutions and supported by the U.S. National Institutes of Health as part of the Clinical Translational Science Award (CTSA) program. ResearchMatch has a large population of volunteers (∼ 150, 000) who have consented to be contacted by researchers about health studies for which they may be eligible. Review and approval for this study and all procedures was already obtained from the IRB of Princeton University (Protocol #15118). We will reach out to 1500 volunteers on ResearchMatch per day. ResearchMatch will release participants’ contact information if they indicate that they are interested in the study. We will then email them with a link to the screener survey.

Our online recruitment, study, and intervention approach will help reduce access barriers for individuals from disadvantaged or minority groups who are less likely or less comfortable engaging with standard health care services (48), thereby reducing biases in the final algorithm.

## Methods: assignment of interventions

### Randomization

#### Sequence generation (21)

We will not generate a sequence in advance. Instead, once participants complete TB2, our automated system will make a random assignment of groups using a python function (i.e., random.choice[‘BA’, ‘CR’]), which returns a random value from the list. We will use a simple randomization and not apply any stratification or restrictions, because we expect unintended differences between groups to be unlikely due to the large sample size of over N = 700 per group.

#### Allocation concealment mechanism (22)

As participants self-enroll, allocation concealment for staff that enrolls participants is irrelevant in our study.

#### Implementation (23)

All major components of the study will be fully automated (see Section 26, *Data management*, for a detailed description of the automated state machine that will govern participants’ progression through the study). The following sequence describes the automated study procedures: Participants will first complete a screening questionnaire. Those who meet eligibility criteria will automatically receive an email invitation to the main study. After providing informed consent, they will receive access to the baseline assessments. Upon completion of all baseline measures (QB1, TB1, TB2; **Figure 1**), participants will be assigned to the CR or BA group using a random number generator on the central computer. This assignment will be recorded in the study database linked to the state machine that defines each participant’s path through the study, and in a separate backup document. Immediately after randomization, participants will receive automated instructions via email specifying which module of e-couch to complete for week 1. In each subsequent week, they will receive further automated emails with instructions on any assessments to complete and which module of e-couch to complete. After completing QB2, participants will be informed regarding which intervention group they are assigned to. All information stored in the database and on the study server will be backed up daily through automated IT processes. The backups will not be accessible by members of the research team. In addition, all email communication will be saved in a log file and backed up by IT processes on separate Princeton servers.

As such, no personnel will be involved in manually enrolling participants, assigning them to interventions, or delivering the interventions. The only aspects of the study that will be handled manually are identity verification of participants, payment processing (i.e., manually sending Amazon gift cards to participants based on calculations from our automated system), and responding to participant-initiated emails or phone calls (e.g., extension requests, troubleshooting, or clarification questions). Personnel cannot manually enroll participants or assign them to groups. If study team members were to make manual changes *post hoc*, these would lead to mismatches across our backed-up records.

### Blinding (24)

Because e-couch is a fully automated online self-help program, there is no direct contact with care providers, and the software treats all participants identically. Consequently, the “care provider” can be regarded as blinded as the program has no bias toward any intervention. All outcomes (questionnaire scores and task performance) are self-reported by participants through assessments automatically delivered through the state machine. Participants will be blinded with regard to their group allocation during all baseline assessments (up to and including QB2), but not for subsequent assessments (including the outcome assessments). Researchers are not blinded during training-data analysis, but the analysis code for the test data will be preregistered before the test set is accessed, ensuring blinded analysis of the test data. Since the intervention is automated, no additional procedures are needed to achieve or maintain blinding. Unblinding does not apply in this trial because participants know they are using e-couch, and no situation requires revealing additional allocation information.

## Methods: data collection, management, and analysis

### Data collection method (25)

Participants will answer self-report questionnaires and complete behavioral tasks at several time points in the study through a website hosted on a secure encrypted server at Princeton University. Data about participants’ engagement with and adherence to the self-help tool e-couch will be automatically collected by the e-couch platform. Thus, all data will be collected automatically without involvement of an experimenter or clinician.

### Socio-demographics data

We will collect self-report data on participants’ age, sex, gender, height, weight, ethnicity, race, sexual orientation, living situation, parental status, education level, household income, marital status, employment, and occupation.

### Self-report questionnaire data

Participants will complete batches of validated self-report measures at several timepoints throughout the study (see section 18, *Participant Timeline* for details). The measures are organized below by conceptual domain:

#### Clinical symptom measures

- Depressive symptoms: Patient Health Questionnaire (PHQ-9; 35) and Quick Inventory of Depressive Symptomatology (QIDS; 49).
- Anxiety symptoms: Generalized Anxiety Disorder 7-item Scale (GAD-7; 50).
- Other mental health symptoms: Mood Disorder Questionnaire (MDQ; 51), Primary Care PTSD Screen (PC-PTSD-5; 52), Alcohol, Smoking and Substance Involvement Screening Test – Lite (ASSIST-Lite; 53), and Peters Delusion Inventory (PDI; 54).

#### Functional impairment and quality of life

- Functional impairment: Work and Social Adjustment Scale (WSAS; 55).
- Quality of life: World Health Organization Quality of Life-BREF (WHOQOL-BREF; 56).
- Well-being: WHO-5 Wellbeing Index (WHO-5; 57).

#### Motivational and reward-related measures

- Behavioral, social, and emotional apathy: Apathy-Motivation Index (AMI; 58).
- Sensitivity to a broad range of rewards: Positive Valence Systems Scale (PVSS; 59).
- Hedonic capacity: Snaith-Hamilton Pleasure Scale (SHAPS; 60).
- Activation and avoidance: Behavioral Activation for Depression Scale (BADS; 61).

#### Cognitive and personality measures

- Maladaptive thinking patterns: Dysfunctional Attitude Scale (DAS; 62).
- Trait worry: Penn State Worry Questionnaire (PSWQ; 63).
- Neuroticism: NEO Five-Factor Inventory, Neuroticism subscale (NEO-FFI; 64, as used by 65).

#### Social and contextual measures

- Stressful life events: Social Readjustment Rating Scale (SRRS; 66).
- Social support: Multidimensional Scale of Perceived Social Support (MSPSS; 67).

#### Therapeutic process measures

- Readiness to change: Readiness for Therapy Questionnaire (RTQ; 68).
- Working alliance: Session Alliance Inventory (SAI; 69).
- Past exposure to therapeutic techniques: Multitheoretical List of Therapeutic Interventions (MULTI-30; 70).

Note that the SAI and Multi-30 will only be asked if participants indicate that they have engaged with a psychotherapist in the last four months at baseline or since the start of the study.

Any modifications of these standard and validated questionnaires (see citations for details of validation and reliability) are listed in the **Supplement *Adaptations of standardized questionnaires***.

#### Study-specific measures

In addition to the validated instruments described above, we will add several study-specific items and questionnaires for assessments of symptoms and treatments where no suitable validated measures were available (see **Supplement *Study-specific measures*** for full item sets). The following self-created measures will be administered:

- Past depression questionnaire: Assesses the onset and duration of depressive symptoms. It will include three free-text questions asking participants to report (a) age of onset (i.e., first depressive episode lasting at least two weeks), (b) total cumulative months of depression experienced during their lifetime, and (c) duration (in months) of the current depressive episode, if applicable.
- Study motivation questionnaire: Assesses participants’ motivation for study participation. It will include four items rated on a 0–100 slider scale (“to improve mood”, “to earn money”, “to contribute to research”, “to improve well-being”) and one open-ended free-text question asking participants to describe their motivation and expectations in their own words.
- Pain questionnaire: Captures recent and chronic pain experiences. It will ask participants about pain occurrence, duration, and location of pain experienced in the past week and of pain that has lasted longer; pain intensity, unpleasantness, and interference with activities using visual analogue scales (VAS; 71); and causes of pain, if known (e.g., injury, surgery, infection, or childbirth).
- Psychiatric treatment questionnaire: Assesses participants’ history of psychiatric treatment (e.g., diagnoses, prior psychotherapy or medication use) as well as their plans for seeking future treatment. Time frames will differ across assessment points: at baseline, participants will report any mental health treatment started during the past four months; at end of the main study, any treatment started since the start of the study; and at each follow-up, any treatment initiated since the end of the main study.
- Return of symptoms questionnaire: Asks participants to rate whether their well-being and mood in the most recent two weeks are better, similar, worse, or otherwise different compared to that at the beginning of the study.

### Behavioral tasks data

Participants will complete several behavioral tasks to assess their learning and decision-making characteristics.

#### Effort task

This task examines effort-based decision-making. On each trial, participants choose between exerting lower effort (fewer button presses) for a smaller reward or exerting higher effort (more button presses) for a larger reward. The required number of button presses varies between 15 and 90, and the associated reward ranges from 3 to 7 points. Each point is worth $0.01 and contributes to a monetary bonus paid at the end of the main study.

Participants are asked to indicate their choice within 5 seconds and then have 40 seconds to complete the required number of button presses. As they do so, they see a balloon filling up, such that it pops (visually, no auditory sound) when they have completed the required number of presses. Across trials, different combinations of effort and reward offered allow us to estimate how participants trade off potential rewards against effort costs.

Reaction times during effort execution provide a measure of effort experience (in contrast to effort anticipation during the decision-making phase). In a subset of trials, participants also rate “How pleasant do you find the reward right now?” – a proxy for subjective reward experience (as distinct from reward anticipation during decision-making).

The task includes 75 trials in total. On two-thirds of the trials, participants execute the chosen effort and receive the corresponding reward. On the remaining third of the trials, after the choice the trial terminates with no effort spent or reward achieved, and the next trial commences. The entire task takes about 18–22 minutes.

The task we will use is an updated version of the paradigm originally described by Berwian et al. (28), where the associated computational modeling approach is also detailed. We conducted a pilot study (*N* = 39) with effort levels ranging from 20–130 button presses to evaluate the one-week test–retest reliability of this updated version. Results demonstrated high reliability across measures: reward and effort anticipation (*ρ* = 0.87 and *ρ* = 0.82; both inferred from computational modeling), reward experience (*ρ* = 0.82; average pleasantness ratings), and effort experience (*ρ* = 0.87; mean button-pressing speed during effort execution). Split-half reliabilities further exceeded these values, as did model parameter recoverability.

Importantly, the correlation between effort and reward anticipation parameters was low (*ρ* = 0.16) and not statistically significant, indicating that these constructs may capture distinct aspects of decision-making. For the present study, we used lower effort levels (15–90 button presses), as prior data from individuals with depression showed floor effects when higher effort levels were used.

#### Scream task

This task uses a fear-learning paradigm comprising four phases: fear acquisition, extinction, spontaneous recovery test, and relearning. The paradigm assesses participants’ ability to learn and update associations between neutral cues and aversive outcomes.

On each trial, participants view one of two visual stimuli (a cartoon image of a moon or of candles) and press the space bar to reveal the trial outcome. Depending on the phase, one stimulus (the moon) is followed by an aversive but tolerable auditory scream on a subset of trials (e.g., 50% during acquisition). Participants wear earphones and complete an individualized calibration procedure before the task begins to ensure the sound is loud but tolerable.

Every few trials during the task, participants are asked to provide subjective ratings. On average, every three trials they rate how likely they expect each stimulus to be followed by a scream, and every ten trials they rate how unpleasant, anxious, or fearful each stimulus makes them feel.

The task proceeds through the following phases: (1) acquisition of the stimulus–outcome association (scream on 8 of 16 moon trials), (2) extinction, during which the screams are omitted, (3) a second extinction test to assess spontaneous recovery of fear, and (4) relearning (scream on 4 of ten moon trials). Each extinction phase was preceded by a break; the first was brief and included completion of the Mini-IPIP Scale (72), whereas the second was longer and involved completing a different behavioral task (the risk task, described below).

The task includes ~100 trials in total and takes approximately 25 minutes to complete. Computational models of this task are described in Berwian et al. (73). Because the task design may induce structure learning, developing a reliable test–retest version is challenging; therefore, this task will be administered only once, before the intervention phase.

#### Risk task

This is a two-armed bandit task designed to reliably estimate learning from positive and negative prediction errors. On each trial, participants choose between a face-up card that consistently yields 5 points and a face-down card that yields either 10 points or 0 points. The face-down cards belong to different “decks”, each associated with a distinct probability of yielding the 10-point reward (20%, 40%, 60%, or 80%).

Regardless of the choice, the face-down card is then revealed, such that on every trial participants receive feedback about the outcomes of both their chosen option and the unchosen alternative (counterfactual feedback). Across trials, this structure enables estimation of how participants update their expectations based on rewards and losses.

There are 16 decks in total, each visually distinguished by a different animal image on the back of the cards. The task comprises 240 trials and takes approximately 14 minutes to complete. Computational models applied to this task, along with reliability estimates, are described in Zorowitz (74).

#### Pavlovian Go/No-Go task

This task is designed to reliably measure Pavlovian biases and their influences on instrumental actions. In this task, participants are shown different ‘robot’ stimuli. Each robot travels down a conveyor belt into a ‘scanner’. Once a robot is in the scanner, participants have 1.5 seconds to either press the space bar (active ‘Go’ response) to ‘repair’ the robot or they can choose to not press any key (‘No-Go’ response).

Participants are informed that they will encounter different types of robots and that their goal is to learn the correct response (Go vs. No-Go) for each type using the feedback (points won/lost) they will receive following each action. Robot types, visually distinguished by different rune images on the robots’ breastplates, each belong to one of 4 categories that vary along two dimensions: the payout domain (gain versus loss) and the correct action (Go versus No-Go). The payout domain is explicitly signaled to the participant by a blue (gain) or yellow (loss) scanner light once the robot is in the scanner, while the correct action must be learned through trial and error (via feedback).

In gain-domain trials, participants can earn payouts of either +10 or +1 points, and in loss-domain trials, participants can lose either −1 or −10 points. When participants make the correct action, they receive the better of the two possible payouts with 80% chance (and the worse payout otherwise). Conversely, incorrect actions yield the worse payout with 80% chance and the better one with 20% chance. This task has 240 trials and takes approximately 16 minutes. For details on the computational models fit to this task, and the psychometric properties of the task, including parameter recovery, test-retest and split-half reliabilities, see Zorowitz et al. (75).

#### Explicit to implicit generalization task

We designed this task specifically to investigate participants’ ability to generalize information learned through explicit cognitive processes to behavior influenced by implicit knowledge. The structure of the task was inspired by Kurdi et al. (76), but updated to include stronger emotional effects and the simulation of a ‘cognitive mini-intervention’ in the middle of the task.

In the task, participants first learn two sets of stimuli (specifically, made-up words ending in “-lapp” or in “-niff”) and their corresponding point values and emotional valence. Learning occurs through repeated choices between pairs of stimuli, each belonging to one of the two categories. Each choice leads deterministically to a horizontal or vertical bar and then to a point outcome (+5 or −5 points) as well as a similarly positively or negatively valenced image, shown for 6–8 seconds. Participants are instructed to focus on the image and fully experience the emotion it elicits (e.g., dolphins playing for a positively valenced image and a punctured knee under surgery for a negatively valenced image).

Participants complete a minimum of 25 and a maximum of 50 of such learning trials including 6 forced-choice trials (three of which lead to negative images). In the remaining non-forced trials, the image shown depends on the participant’s choice. Knowledge of the learned associations is then assessed through 10 test trials in which participants again choose between words from the two categories, but no feedback is provided.

Next, to simulate a ‘cognitive mini-intervention’, participants are explicitly informed through written instructions that the relationship between the horizontal and vertical bars and the outcomes has reversed - the bar that previously led to positive outcomes will now lead to negative outcomes, and vice versa. After reading the instructions, participants are asked to take time to consider these changes while seeing the instructions for another 30 seconds. Then the explicit knowledge test is repeated. Following this, a modified version of the Implicit Association Test (IAT; 77) is used to measure participant’s implicit evaluations of the stimuli based on their reaction times. Trials in this phase involve responding to stimuli (one of the previously seen words or emotion-inducing images) with the left or right keys according to one of two mappings – “-niff/positive” on the left and “-lapp/negative” on the right, or, in other blocks, “-niff/negative” on the right and “-lapp/positive” on the left. Faster response times indicate congruence between the judgment of the valence of the word and the valence of the images categorized to that same side.

This task has about 150 trials and takes approximately 17 minutes to complete. We extensively piloted this task to obtain a version in which more than 80% of the participants achieve accurate reversal on the explicit knowledge test, while showing substantial individual differences in the implicit association test. Due to the nature of the task with a reversal in the middle, testing for test-retest reliability is not trivial due to structure learning. For this reason, the task will be administered only once, prior to the start of the e-couch intervention.

#### Symmetry span task

This task is designed to measure working memory capacity (78). Participants will be shown a sequence of 2–5 red squares presented one at a time within a 4x4 grid. They are asked to recall both the locations of the squares and the order in which they appear. After each square presentation, participants complete a second task: judging whether a black-and-white pattern displayed in an 8×8 grid is symmetrical along its vertical axis. This judgment is made using the left or right arrow key before the next red square appears. Once the sequence is complete, participants recall the sequence by clicking the corresponding grid locations in the correct order. The task has 40 trials and takes approximately 10 minutes to complete. For validation and reliability of the task, see Oswald et al. (78). Note: version 1.0 of this protocol did not include this task.

#### Matrix reasoning task

The matrix reasoning task (79–81) will be included as a measure of general cognitive ability and to index attentional engagement across tasks. In this task, participants must solve a series of puzzles. On each trial, participants view a 3x3 matrix of abstract shapes, with one cell (the bottom-right) remaining empty. Participants are instructed to complete the matrix by identifying the missing element from four possible alternatives. To do so, participants must infer the underlying logical pattern, such as changes along the vertical, horizontal, or sequential dimensions, based on the arrangement of the other shapes. No feedback regarding correctness is given. The task has 12 trials and takes approximately 3 minutes to complete. Zorowitz et al. (81) reported good reliability and convergent validity of an open-access item bank of matrix reasoning tasks in large online samples, which is the version we will use in this study. We use different sets of stimuli when we repeat the task at different time points in the study (note: version 1.0 of this protocol used the same set of stimuli multiple times).

### e-couch platform usage data

Participants who complete baseline assessments and meet all eligibility criteria will be issued a unique login token, providing them with free access to the e-couch platform. These tokens will allow participants to create an account and engage with the e-couch intervention modules.

Cumulative usage data associated with each participant’s token will be collected throughout the study period. We will manually download these data from the e-couch administrative platform approximately once per week. Each download will include updated cumulative usage information for all participants.

Examples of the data to be extracted include general platform activity (such as number of logins, registration and last login dates, total time spent on the platform, and first and last access dates), as well as module-specific engagement metrics (such as progress status, time spent within each module, and number of accesses). These data are automatically logged by the platform and reflect participants’ lifetime engagement with the intervention. While additional variables are available through the platform, only a predefined subset relevant to the study will be extracted, and not all extracted variables will necessarily be used in the final analysis.

As the data are system-generated, no assessor training is required. The reliability and validity of the usage data are determined by the platform’s internal tracking mechanisms. A codebook detailing all available data fields is maintained by the platform provider and can be made available upon request.

### Processes to promote quality of data collection

To promote data quality, we will implement several procedures, including attention checks, identity verification, and study engagement checks. Participants will be excluded if they demonstrate inconsistencies in their self-report information during the identity check, show inattentive task performance, or lack engagement with the study (e.g., fail to provide timely replies to study emails). These procedures are described in detail below.

#### Attention checks

We will implement several attention checks within the self-report questionnaires and will employ the Matrix reasoning task as a way to measure random task behavior. We will have three attention-check questions in two self-report questionnaires (PHQ-9 and WHOQOL-BREF) at the beginning of the study. For example, we will ask “*How well are you able to touch the peak of Mount Everest with your hands right now?*”, where participants are expected to only answer “*Not at all*”. We will also use task behavior in the matrix reasoning task to assess whether participants are paying attention during the tasks. If participants answer fewer than three answers correctly in the matrix reasoning task (i.e., below chance level), or respond to more than six questions in 3 seconds (suggesting they are not thinking the problems through), they will be marked as failing the matrix reasoning task. If participants fail more than one of four (three attention check questions and matrix reasoning task) attention checks within the first five minutes of the study, they will be excluded from further participation in the study. Participants will be notified that if they believe they had been excluded by mistake or due to technical issues, they can contact us for an identity check and to explain the situation. We will invite them back to the study upon successful completion.

#### Identity check

Participants who successfully pass the attention checks may be invited to a brief zoom call with the experimenters to verify their identity. We will randomly invite approximately 20% of participants recruited through ResearchMatch (given that in our experience they are a reliable recruitment platform) and all participants recruited through other channels. Once invited, participants will have two weeks to book a time-slot for the 5-minute zoom meeting. During the zoom meeting, we will ask participants for basic information they had provided in the screener or demographics questionnaire and compare their verbal answers to the ones previously provided. We will also ask them to show a photo ID to the camera for verification of information such as location and age. We will exclude participants from the study if there are multiple mismatches between the information they provide and their screener responses, or if they fail to schedule a zoom meeting within the required timeframe. In these cases, we will also withhold payment for their time in the study so far, as we cannot verify their identity in order to determine whom we should compensate. Note: this procedure is updated from protocol version 1.0, which did not include identity checks (see **Supplement *Protocol changes***).

#### Study engagement

At any time, we may exclude participants from future portions of the study if they show a significant lack of response and/or involvement in the study components. For example, if a participant does not complete required study components in a timely manner after receiving email reminders, we will inform them that their participation in the study has been discontinued. In such cases, we will compensate participants for all portions they have already completed. If they had already received a token to access e-couch, the token will remain active, and they can continue using e-couch at their discretion.

#### Additional attention checks

We will incorporate task-specific attention checks in each behavioral task to flag participants who did not pay adequate attention in that task. We will perform sensitivity analyses with and without these participants (see section 27d, *Methods for any additional analyses*). The flagging and potential exclusion criteria for analysis of each task are as follows:

- Scream task: The scream task includes six audio attention checks in which participants are quietly instructed to press a specific letter key (e.g., “Please press the letter G now”). The purpose of these checks are to ensure minimum audio volume as participants who decreased the volume substantially will not hear the scream. Participants will be excluded if they fail more than two attention checks.
- Effort task: Participants will be excluded if they timed out in more than 10% of the trials. A trial times out if the participant does not make a choice between the two options within 5 seconds or fails to complete button pressing within 40 seconds on trials requiring effort execution; all timed-out trials are repeated, however, timing out on more than 10% of trials will be grounds for exclusion.
- Risk task: Participants will be excluded if they require more than five attempts to complete the comprehension check, do not show different risky choice probabilities for the 20% and 80% reward cues or fail to exhibit choice sensitivity to winning 10 versus 0 points.
- Pavlovian Go/No-Go task: Participants will be excluded if they exhibit chance-level performance (*<* 55% correct responses) on go-to-win trials.
- Explicit to implicit generalization task: Participants will be excluded if they do not achieve 80% correct response in the initial learning phase.

In addition to initial questionnaire attention checks within the first five minutes of the main study, we will include questionnaire attention checks throughout the entire study to assess data quality and to ensure that participants are reading questions carefully and not replying randomly to self-report instruments.

### Plans to promote participant retention and complete follow-up, including list of any outcome data to be collected for participants who discontinue or deviate from intervention protocols (25b)

We will encourage participants to complete the entire study, including all three follow-up questionnaires, by providing a $10 bonus upon completion of the final follow-up. Participants who withdraw before completing the study will also not receive performance bonuses earned from the behavioral tasks. Automated reminder emails will be sent to participants 7 and 14 days after the start of each follow-up. However, participants may complete each follow-up questionnaire up until the subsequent follow-up is sent out. For the final follow-up questionnaire, participants will have up to three weeks to complete it. For participants who choose to withdraw from the study after commencing it, we will document any reasons they provide by email and securely store this information in our database. When available, we will also record reasons for non-adherence (e.g., discontinuing due to burden or lack of interest) and non-retention (e.g., withdrawal or lost to follow-up). No additional data will be collected from participants who withdraw from the study.

### Data management (26)

We developed software for an automated state machine to track each participant’s progress in the study and to manage study-related email communications. The system sends emails with invitations to specific parts of the study or the self-help tool, as well as reminder emails, based on completion of previous sections and time lags. The state machine runs on a secure server with encrypted file systems. Access is restricted to authorized study members working on this project and IT staff, all of whom must use two-factor authentication. The system records participants’ progress. Eligible participants identified in the screener will receive automatically a main study invitation, while ineligible participants will receive an email informing them that the study is not a good fit for them.

The state machine tracks how long a participant remains in each part of the study and logs every time point at which a participant starts or completes a task or questionnaire set. It also sends reminder emails to participants who do not complete task or questionnaire batches within the allotted time and automatically excludes participants who exceed the grace period, as detailed in section 15c, *Strategies to improve adherence to intervention*. The software also performs random assignment of participants to intervention and training/test groups and distributes e-couch tokens.

We note that the above steps occur automatically, without any involvement or influence from study team members. However, study team members may manually adjust a participant’ progress in the study if the participant requests to skip a task or ask for an extension. Such requests, as well as communication with participants beyond answers to clarification questions and providing payment information, will be documented in the database described below. Any complaints or events requiring IRB involvement will be documented and reported to the IRB through Princeton’s electronic IRB system (eRIA).

The software maintaining the state machine is documented in version-controlled, time-stamped Git repositories. The code includes detailed definition on which data is stored when and how. We also maintain a log of all server updates, including timestamps of new source code commits, their correspondence to IRB-approved protocol modifications and their launch on our server. All these information can be accessed by request. We use Alembic, a database migration tool, to track and apply structured updates to the study’s database, ensuring that all changes to its structure are versioned, documented, and reproducible over time.

#### Data storage and security

We will save two types of data separately. First, we will save metadata in a PostgreSQL database stored on the same encrypted server as the state machine. This will include all data generated by the software that runs the state machine (e.g., timestamps and completion logs). The metadata will also contain participants’ email address, participant IDs, group assignment information (i.e., BA or CR, and training or test), task and questionnaire batch access links, e-couch tokens, identity check assignments and reasons, withdrawal or exclusion reasons, consent information, and documentation of completion of task and questionnaire batches (including individual tasks), each with an associated timestamp. A subset of the metadata information is also saved in participants’ individual metadata files.

Second, we will save data from self-report questionnaires and behavioral tasks in time-stamped files after completion (or early termination) of each task or questionnaire batch. Each file will include the participant’s participant ID and the name of the relevant task or questionnaire batch. Data from training and test groups will be stored in two separate locations throughout the study.

Task and questionnaire data will be stored on both the primary encrypted server and on a second, separate Princeton server that is not accessible via the Internet. We will routinely verify that all task and questionnaire data have been successfully saved to the second server; once verified, we will delete the corresponding task and questionnaire data files from the primary server that is connected to the internet, as a precautionary data-security measure in case the primary server were compromised. We will additionally use command line tools to back up task and questionnaire data to another separate location on the second server.

Data collected while participants engage with the e-couch program will be stored on e-couch’s secure servers in accordance with their privacy policy https://ecouch.com.au/info/privacy. We will download pseudonymized usage data (that is, identified only by participants’ e-couch tokens) from the e-couch administration interface weekly. These data will then be transferred to Princeton University’s secure servers.

We will advertise and recruit participants via ResearchMatch (see section 20, *Recruitment*). Email addresses of participants who express interest in our study on ResearchMatch can only be exported jointly with other information they provided to ResearchMatch, such as name, address, demographics, veteran status. ResearchMatch requires that these data are only saved on encrypted devices. Accordingly, we will download and save these data directly to an encrypted device. We will then delete all information except the email addresses, which are needed to send participants the study information and a link to the screener.

### Statistical methods (27)

The goals of our study are multifaceted. First, we aim to build algorithms that predict who would benefit from CBT-based interventions for depression, and specifically whether behavioral or cognitive interventions would be more beneficial for a given individual. Second, we are interested in testing whether learning and decision-making capacities assessed through our behavioral tasks improve the predictive accuracy of these algorithms – if so, this would suggest that the cognitive mechanisms assessed through the tasks may be related to the processes involved in CBT. Finally, we seek to uncover how these learning and decision-making capacities influence (i.e., moderate) participants’ responses to the interventions and whether changes in some capacities mediate the responses. In developing these algorithms, we aim to ensure that their application is feasible in a real-world clinical setting, requiring minimal additional time or resources. We also seek to ensure that our results are as reliable and robust as possible.

One challenge we face with these goals is that no prior evidence exists regarding our task-derived measures, their predictive power for differential outcomes of BA and CR, and their variation in interactions with clinical and sociodemographic variables. Without such prior data, it is difficult to anticipate the precise nature of these interactions or to identify the most adequate and least biased analytical approach.

A further challenge arises due to different adherence levels across participants which dilute moderation effects and weaken differential prediction effects in intention-to-treat (ITT) analyses. To address this, we plan to incorporate adherence levels into our models and adjust for potential biases that may arise (see Section 27d, *Methods for any additional analyses*). We are not aware of any study in the mental health literature that has included predicted adherence scores in individual-level differential prediction models, but will expand on relevant prior work suggestions in the literature (82).

To address these challenges while ensuring that our final results are not influenced by different types of data leakages, we will randomize participants into training and test datasets (2:1 ratio) at the time of consent to the main study. This will allow us to conduct a series of exploratory analyses on the training dataset and finalize our precise analytic approach using power and sensitivity analyses.

We will then preregister all hypotheses, algorithms and a code pipeline before accessing and applying them to the test dataset.

While our test dataset does not constitute a fully external dataset, as highlighted in guidance on prediction model evaluation and generalizability (83, 84), this approach has the following advantages given our dataset size:

- Preventing data leakage, which often occurs with improper cross-validation on a single dataset (85).
- Allowing for tuning of hyperparameters such as decision thresholds.
- Allowing to explore more advanced statistical methods that can then be validated.
- Safeguarding against false positive results.
- Ensuring that researchers’ decisions based on observing the training data do not result in bias or overfitting of the test data.
- Replication of results.

Our preregistration will include all required details including, among others, the statistical approach, effect size, significance levels and adjustments, as well as code implementing these.

We acknowledge that our two-step approach with a detailed pre-registration of the analysis plan for the held-out test dataset done after data collection (but prior to observing these data) is non-traditional in the context of clinical trials. However, given the complexity of the statistical approach we are taking, the size and thus power of our dataset, the timeline we have to collect the data, and a setup where we can ensure that the held-out test data are not observed by a human during the study, we believe that this is the most useful methodological approach we can employ to achieve robust results and inform subsequent research.

We outline below the most appropriate approaches we have so far identified for our exploratory analyses for our main research questions. Because of the rapid evolution of the literature and recommendation on analysis methods we plan to conduct, we may use a different approach if it is clear that a more suitable and less biased approach is available at the time of analysis of the training data.

### Statistical methods for primary outcomes (27a)

#### Preprocessing of data

We will compute summary scores for all questionnaires in accordance with scoring procedures outlined in the original validation papers (see Section 25, *Data collection method* for the list of questionnaires and references). For behavioral task data, computational models will be applied where appropriate to infer underlying cognitive mechanisms and enhance measurement reliability (see Section 25, *Data collection method* for task descriptions and citations of papers describing the computational models corresponding to each task). Our computational modeling approach for task data includes model comparison, parameter estimation, model recovery, parameter recovery and posterior predictive checks of the winning model.

All data preprocessing steps will be treated as components of the modeling pipeline and performed within each training fold only, to prevent information leakage. Continuous predictors will be centered and scaled using parameters estimated from the training fold and then applied to the corresponding validation fold. Missing predictor data will be handled via multivariate imputation by chained equations (MICE), with imputation models fit within training folds only, as described in section 27c, *How missing data will be handled*. Because the study is not multi-site, no harmonization across sites is required.

We will preregister the code for all preprocessing steps before applying them to the test dataset.

#### Primary analyses: predicting response to CBT-based interventions

Our primary analytic goal is to predict who will benefit from CBT-based interventions and who might benefit more from BA versus CR. In line with recommendations by (84), predictors will be exclusively based on variables collected during the baseline assessment and their definition or computation will not be dependent in any way on outcome variables. Similarly, outcomes will be defined independent of any predictor variables (e.g., we will not use change scores as outcomes).

Model development will be conducted exclusively within the training data using an internal validation framework. Specifically, we will use nested, repeated 10-fold cross-validation, with the full procedure repeated 100 times to improve the stability of performance estimates. Identical cross-validation splits will be used across all candidate models to enable fair model comparisons.

To develop our models, we will first perform systematic variable selection and exploratory modeling on the training dataset using cross-validation to assess consistency and prevent overfitting. We will add to the models groups of related predictors (such as demographic variables, clinical variables, task-derived variables) that would be presumably assessed jointly in a clinical setting and subsequently use automated feature selection and regularization to determine which predictors to include in the final model. Predictor selection will be treated as part of the model development process and conducted exclusively in the training data. The finalized predictor set for each model and time point will be fixed and preregistered prior to confirmatory evaluation on the held-out test data.

We will evaluate multiple model classes, including penalized regression approaches (e.g., lasso and elastic net) and random forest models. Hyperparameter tuning will be performed exclusively within the training data using a nested cross-validation framework, with all tuning restricted to inner folds. Performance metrics will include balanced accuracy for classification models and mean squared error and *R*^2^ for regression-based models. Model performance will be summarized using pooled out-of-sample predictions across all cross-validation folds and repetitions. For classification models where AUROC will be used as the primary discrimination metric, DeLong’s method (86) will be used to obtain confidence intervals and to test differences in AUROC between models. Calibration will be performed within the nested cross-validation framework using out-of-fold predictions from the inner loop – that is, using predictions that are generated for participants in the outer-training fold by a model that did not include those participants during fitting, ensuring no information leakage. A calibration model (e.g., Platt scaling for classifiers) will then be fitted to these predictions, where appropriate. We will report mean absolute prediction error and quantify uncertainty around individual predictions. The methods to generate prediction uncertainty will be determined and set during model development and applied without modification to predictions from the held-out test dataset. The finalized modeling pipeline, including the internal validation strategy, preprocessing steps, target optimization metric, and any hyperparameter tuning procedures, will be fixed and preregistered prior to confirmatory evaluation on the held-out test dataset.

Following confirmatory evaluation of the preregistered models, we will conduct *post-hoc* interpretability analyses using SHAP values to aid in understanding the contribution of individual predictors to model predictions (87).

More specifically, we will first create predictive models using only clinical and demographic variables, which will constitute our baseline models. For the preregistration, we will choose one baseline model considering predictive power, stability, clinical feasibility and interpretability. Preregistered analyses of the test data will assess the extent to which this model improves predictive power as compared to chance.

Second, we will add task-derived variables from each task separately to the baseline model to test whether these variables improve the predictive accuracy. For each set of task variables that we find to improve predictive accuracy, we will preregister the corresponding analyses for confirmation in the test dataset. We take this single-task approach as we consider each task as a separate instrument to be tested for efficacy independently, and to improve clinical feasibility (as one 15-minute task is clinically more feasible than a two-hour task battery) and interpretability (as some tasks assess correlated features, so combining them could obscure which variables drive the effects).

We will take this two-step approach both when examining how well we can predict whether an individual will respond to CBT-based interventions, and whether we can differentially predict whether they will benefit more from BA vs CR. For algorithms aiming to predict depression scores at week six after engaging with any CBT-based interventions, the final outcome will be out-of-sample predictive accuracy above chance (for the baseline model) or above the baseline model (for models including task data). For algorithms predicting differential outcome for behavioral vs. cognitive interventions, we will train algorithms for each arm separately. Following the approach of the personalized advantage index (PAI; 88, 89), we will use the model to predict for each participant the outcome for the intervention they received (using models trained on data not including this participant) and for the one they did not receive (counterfactual outcome). The final outcome will constitute whether individuals who were predicted to have received their more beneficial outcome have lower depression scores at the 6-week mark than those who the algorithm predicts would have fared better from the other intervention (and how much that difference improves by adding in task parameters).

#### Uncovering mechanisms of change

To understand in detail how specific task variables relate to changes in symptoms, we will run two types of analyses. We will conduct moderation analyses to examine whether specific variables only influence response to one, but not the intervention. For tasks that were assessed a second time during the interventions, we will use mediation analyses to test whether change in specific task variables by the middle of the intervention can account for the change in symptoms by the end of the intervention.

### Who will be included in the analyses (27b)

For all analyses, we will include only participants who (a) met the assessed inclusion and exclusion criteria specified in Section 14a, *Eligibility criteria*, and (b) passed identity and attention checks at the beginning of the study. For predictive modeling and causal inference, the analytic sample will include participants who were randomized and had a baseline PHQ-9 total score greater than 4. This constitutes our intention-to-treat (ITT) sample.

### How missing data will be handled (27c)

We anticipate a low proportion of missing data from self-report questionnaires and behavioral tasks from baseline as data collection is automated and participants can only advance after completing all components within a batch, i.e., such missing data will be missing completely at random (MCAR). Nevertheless, technical issues may occasionally result in missing data. We will impute missing data for questionnaires and task data using recommended imputation procedures such as Multivariate Imputation by Chained Equations (MICE, 90). Imputation will be applied for predictors and independent variables. As we assume that outcome data might be missing not at random (MNAR), we plan to not impute outcome data. However, we will examine all our assumptions about missingness mechanisms through sensitivity analyses and adjust our imputation decisions and analyses accordingly. During model development, imputation will be performed in a nested manner within the cross-validation framework such that imputation models are fit using only the training data within each fold, with no access to held-out folds. No auxiliary variables beyond the predictor set of the model under evaluation will be included in the imputation models (91). For models that make separate predictions per group, imputations will be conducted separately by intervention group (92). The finalized imputation strategy, including the predictor matrix, will be specified in the preregistered confirmatory analysis plan prior to evaluation on the held-out test dataset.

Participants who wish to do so will be permitted to skip two behavioral tasks, the (*Explicit to implicit generalization task* and the *Scream task*), which involve emotionally aversive stimuli, without withdrawing from the study. As this constitutes a case of structured missingness and imputation of task data would not add information to answer the question whether task data improves the explanatory power for outcome above and beyond baseline measures, we will not impute data for individuals who skipped these tasks. Instead, these participants will be excluded from analyses that directly involve measures derived from these tasks, but will be retained in all other analyses. To assess potential bias associated with task noncompletion, task non-completion will be explicitly coded as an indicator variable, and sensitivity analyses will be conducted comparing results with and without participants who skipped these tasks. Associations between task non-completion and symptom severity will also be examined to evaluate whether avoidance of aversive stimuli relates systematically to clinical characteristics. This is a purposeful design feature that weighs subjective feelings of discomfort of possibly vulnerable participants against building generalizable predictive algorithms. We acknowledge that the generalizability of our algorithms will have to be tested in individuals who prefer not to be exposed to negative emotional stimuli, possibly in an in-person study with more immediate clinical support if the algorithms are demonstrated to be useful. We will explicitly acknowledge in the publications of results related to these two tasks that findings may not generalize to individuals with higher avoidance or lower distress tolerance, and discuss this as a limitation.

In contrast, several questionnaires (*NEO-FFI*, *PSWQ* and *MSPSS*) and the *Symmetry* sp*am* task were introduced after the first 100 participants (in the transition from protocol version 1.0 to version 1.1 described here) and thus were not available to the initial cohort. For these tasks, all participants will be included with imputed data if data are missing.

For confirmatory analyses on the test dataset, missing-data procedures will be finalized and preregistered before accessing the held-out test dataset to ensure analytic independence.

### Methods for any additional analyses (27d)

#### Sensitivity analyses

For all cases where we have to exclude participants, we will report the means and distributions of the baseline characteristics of included and excluded participants. To assess whether the change in inclusion and exclusion criteria from protocol version 1.0 to 1.1 influenced our sample or results, we will compare baseline characteristics of participants included before and after these changes and compare models trained with and without participants included under protocol version 1.0. Similarly, through sensitivity analyses in the training dataset, we will compare models with and without participants who meet the exclusion criteria in the learning tasks. We will also attempt to impute data from participants who were excluded due to inattention or, alternatively, use failed attention check as a variable that interacts with all other task variables and base our pre-registration on these results to adhere as closely as possible to our intention-to-treat analyses. We will apply a range of sensitivity analyses to test the assumptions and effects of imputations such as: comparing analyses with imputed values to complete-case analyses (without imputed values), testing for bias due to data that are missing not at random (i.e., adding delta adjustments) and testing for robustness of results across different numbers of imputed datasets that are averaged over (93).

We will additionally assess outcome distributions in the exploratory model-development dataset prior to model fitting. For binary outcomes, we will quantify class imbalance and evaluate performance using balanced accuracy. When a substantial imbalance is observed, we will conduct sensitivity analyses using imbalance-aware approaches (e.g., class-weighted models and synthetic oversampling, where appropriate), applied only to the training data of each resampling iteration to avoid data leakage. For continuous outcomes, we will examine distributional characteristics and evaluate model performance using mean squared error and *R*^2^. All outcome-distribution related decisions will be made using the training dataset; the held-out test dataset will not be used for outcome balancing or model tuning.

Of note, we hypothesize that at least some of our examined variables will moderate the effect of the intervention on the outcome. However, such moderation can only take place if participants actually adhered to the assigned interventions. We will explore several approaches to account for adherence in our modeling and preregister the most adequate approach for the test dataset. If possible given our sample size and data structure, we will jointly model adherence and intervention outcome or build a model to predict adherence and use the predicted adherence level in the model that predicts outcome or stratify participants based on the predicted adherence levels. This will account for the fact that adherence cannot be measured clinically prior to selecting a treatment option. At minimum, we will run our analyses as ITT analyses to measure the effect as per-assignment (which is closely related to the clinical case) and separately as per-protocol analyses (only including participants who adhered to the assigned interventions), to assess the effect of adherence on predictive and causal effects. In the latter case, we will adjust for possibly breaking the randomization in adherers with recommended strategies from the literature (94). To evaluate algorithmic fairness and generalizability, we will conduct subgroup analyses comparing predictive performance across sociodemographic groups (including race, gender identity, and age bands), with descriptive statistics reported for smaller subgroups where formal statistical comparisons may be underpowered. In the latter cases, we will acknowledge that the lack of ability to examine the generalizability is a limitation of the current study.

## Methods: monitoring

### Data monitoring committee (28)

Our IRB and our sponsor do not require a data monitoring committee for this type of study, since we are using an existing thoroughly tested and publicly available self-help intervention. Due to the use of this tool and the fact that our study procedures do not exceed minimal risk, no stopping guidelines are required.

Members of the study team will conduct data monitoring. After the first 100 participants (all assigned to the training dataset), we conducted data quality analyses. These analyses indicated that a substantial proportion of the participants showed random task behavior, failed our attention checks or may have enrolled in the study multiple times under different identities (likely to obtain the participant compensation several times). As a result, we updated the eligibility and exclusion criteria from protocol version 1.0 to exclude participants who fail multiple attention checks or fail the identity check (see section 14a, *Eligibility criteria*, and section 25, *Data collection method*, for details).

### Trial monitoring (29)

We have not scheduled any external monitoring. Because the study poses only minimal risk, neither our sponsor nor our IRB requires planned external monitoring. Each implementation version of our study is saved with a timestamp in a GitHub repository, allowing us to reconstruct any past version for external monitoring at any time, effectively tracking the “live” version’s evolution. Additionally, the server code and data are backed up daily by IT, and can be made available by them for external monitoring. Local investigators will continuously monitor study implementation, data quality and storage, and communications with participants.

## Strengths and limitations of this study

### Strengths

- Automated online assessments and automated intervention delivery avoid biases and enables a large sample size.
- We examine novel and easily assessable predictors (thus, high potential for translation), specifically, learning capacities informed and matched based on theoretical understanding of change processes in the examined interventions.
- Separating data into training and test dataset allows the application of advanced statistical approaches to build – and then validate – a generalizable differential intervention recommendation algorithm.

### Limitations

- As the sample will be recruited entirely online, data quality may differ from that of samples collected in clinical settings.
- The use of low-intensity self-guided interventions may limit generalizability to the clinical setting.

## Administrative information

### Protocol version (2)

Version 1.1.

For changes made to this version compared to version 1.0, see **Supplement *Protocol changes***.

### Name and contact information for the trial sponsor (3b).

The Trustees of Princeton University, 1 Nassau Hall-Princeton University, Princeton, New Jersey, USA, 08544.

### Role of trial sponsor and funders in design, conduct, analysis, and reporting of trial; including any authority over these activities (3c).

The sponsor and funding body have no role in study design, data collection, analysis, interpretation, or in writing of publications. The funding body will check manuscripts prior to publication to ensure adequate protection of any intellectual property.

### Composition, roles, and responsibilities of the coordinating site, steering committee, endpoint adjudication committee, data management team, and other individuals or groups overseeing the trial, if applicable (3d).

The trial steering committee consists of Dr. Yael Niv and Dr. Isabel Berwian. They will make executive decisions and have the day-to-day oversight of the trial implementation.

## Open science

### Trial registration (4)

This study was registered at ClinicalTrials.gov (ID: NCT06631183) under the title “Behavioral Study to Predict the Efficacy of a Self-Help Tool” and first submitted on September 24, 2024. It was posted publicly on October 8, 2024, and can be accessed through https://clinicaltrials.gov/study/NCT06631183. This trial was retrospectively registered (approx. 9 months after enrolling the first participant) because the authors were not aware that prospective registration was required for this type of study. The funder, sponsor and ethics committee of this study did not require prospective registration for this type of study and we (the authors) were not fully aware of the details of the ICJME recommendations requiring prospective prospective registration. As soon as we became aware that prospective registration was required, the trial was registered (which occurred during the enrollment phase). The risk of bias due to late registration is low as the analyses of the untouched test dataset, which constitutes the key dataset, will be preregistered in detail (including analyses code) after analyses of the training dataset and prior to accessing the test dataset. Additionally, our entire study design was automatized and saved in a time-stamped github repository, which can be accessed for verification upon request. This note has been added *post-hoc* to protocol version 1.1 when the protocol was prepared in line with the SPIRIT guidelines.

### Protocol and statistical analysis plan (5)

The protocol will be accessible online as open access publication on the publisher’s website. The statistical analyses plan for the held-out test dataset will be preregistered and accessible on OSF.

### Data sharing (6)

We will share de-identified questionnaire data and behavioral task data with other researchers from the Wellcome Leap consortium. After publication, these same data will be shared with other researchers upon reasonable request, that is, providing that the requester is a non-commercial entity and will treat the data in line with our ethical principles. We will make the data publicly available only if we feel it is possible to ensure the data would be used for the benefit of individuals with mental health symptoms. Participants will be informed about these data sharing plans prior to consenting to participate in the study.

### Dissemination policy (8)

We aim to publish the results of this study in scientific journals in the fields of clinical psychology, psychiatry, clinical practice, and computational psychiatry. We will also share the results via scientific talks and poster presentations at conferences for researchers, healthcare practitioners, and/or the public. Furthermore, we will disseminate the results to the public through various public engagement channels, including online science communication articles, press releases, and social media content.

## Ethics

### Research ethics approval (30)

This study was approved by the Princeton University Institutional Review Board as Protocol #15118.

### Protocol amendments (31)

Decisions on any changes to the study protocol, including modifications to participant communication, study procedures, study tasks or questionnaires, and eligibility criteria, will be made by the trial steering committee. Proposed modifications will be reviewed and approved by the Princeton University IRB prior to implementation.

### Consent or assent (32)

Participants will complete two separate online consent forms: one before the screening survey that applies only to the screening survey procedures, and, if they are eligible for the study and choose to participate in it, another consent form before beginning the main study. Each form clearly outlines the purpose, procedures, potential risks and benefits, data confidentiality, and the voluntary nature of participation in the relevant portion of the study, using plain language. CITI-certified researchers, including PI Niv, will prepare and monitor the consent process and will be accessible by email or phone (mentioned on the consent forms) to answer any questions participant may have before they give their consent. Consent will be obtained electronically via a secure, encrypted Princeton University server that can be accessed only by the study’s research team through two-factor authentication. Only participants who actively select all consent options will proceed to the study procedures. No ancillary studies are planned at this time; if any arise, participants will be re-contacted to obtain additional consent for any use of their data beyond the original agreement.

### Confidentiality (33)

This research is covered by a Certificate of Confidentiality from the NIH. Researchers with this certificate will not disclose or use information that may identify participants in any federal, state, or local civil, criminal, administrative, legislative, or other action, suit, or proceeding, even if there is a court subpoena. Exceptions include: a) A federal, state, or local law that requires disclosure, such as reasonable cause to believe that a child has been subjected to abuse or acts of child abuse. b) Participants’ explicit approval for the researchers to release their name and/or personally identifiable information. For information on safe data storage, see section 26, *Data Management*.

### Ancillary and post-trial care (34)

Participation in the study involves minimal foreseeable risk, primarily related to the emotional responses that may be invoked by questions in the self-report questionnaires, or images and sounds presented during the tasks. Some of the visual stimuli involve very disturbing and graphic contents (e.g., an image of a person getting hit by a car, or an image of mummified bodies), which participants may find troubling. In addition, this study involves being exposed to three kinds of screams that may be startling and unpleasant. The study also includes a variety of psychiatric symptom questionnaires. These do not permit formal diagnosis of clinical conditions, however, the questionnaires do assess symptoms associated with psychiatric disorders such as depression and anxiety, including one question that touches on suicidality. The sensitivity of the material might, in principle, be troubling to participants or might bring their attention to issues already of concern. Hence, throughout the study, and especially at each timepoint in which participants are asked to provide mental health information or engage with aversive stimuli, we will provide a participants with a “Mental Health Resource Page” (see **Supplement *Mental health resources page***) and encourage them to seek further help if needed. This is to ensure that if a participant experiences significant distress due to the questions or the task, they have resources for support at their fingertips. We opted to ask about suicidality given that empirical work suggests there is no evidence that asking about suicidal ideation increases the risk of suicide (95), and do not exclude participants based on suicidal ideation to not discriminate against them in a study that can help with such symptoms. Indeed, providing details of access to care might reduce ideation and promote treatment seeking.

Participation in the e-couch program also involves minimal foreseeable risk. The program is designed similarly to a self-help book that is delivered through a website and is interactive in that it prompts participants to do quizzes and make plans to change their thinking patterns and behavior. As stated on the e-couch website, the program is not intended to replace medical treatment, but educates participants about treatment options and encourages them to seek them if needed (https://ecouch.com.au/info/terms; https://ecouch.com.au/info/faq). The program also refers participants to relevant services if they feel they need urgent help (https://www.ecouch.com.au/info/emergency_help). Furthermore, the first module with which participants will engage in our study focuses on psychoeducation, and includes information on when to seek medical treatment for depression. The program has been extensively tested in three randomized controlled trials (36–38). No withdrawal or incidents of adverse events were reported in these papers. Throughout our study, we will not discourage participants from pursuing other treatments and will not exclude them if they take antidepressants or start new treatments. Thus, while receiving psychotherapy or planning to start such will be an exclusion criterion, we will not remind them of that and will not exclude participants if they start psychotherapy treatment during the study. We will inquire about treatments started or changed during the study in the final and follow-up questionnaires, and may use such information in our analyses, however, we will never suggest that seeking or obtaining treatment for symptoms is in any way problematic.

To the contrary, once participants complete all assigned modules, at the end of week 6, we will inform them that they can now use all other modules provided by e-couch (i.e., Interpersonal Psychotherapy, Problem-solving, Relaxation, and Workbook), and list the modules the alternative group (that they were not assigned to) had completed. These design choices are aimed at maximizing the therapeutic benefit that participants may gain from the study.

Participants will be compensated for the time completing tasks and filling questionnaires at a base rate of $15 (USD) per hour, for up to 4 hours of participation (compensation will be calculated for all tasks and questionnaires a participant completed using the estimated time for each, not the actual time spent). Participants will be able to earn up to an additional $14 through bonus points for some tasks, and another $10 bonus for completing all 8 study parts, as well as the e-couch modules as instructed. We will ask participants to confirm their email address within 2 weeks of completion of the main study and will email them the compensation in the form of an Amazon gift card once at the end of the main study ($51 plus task bonuses) and after the follow-up period (up to $10 plus the $10 bonus for completing all study parts). The screening survey will not be paid, nor is time on e-couch compensated, in order to not coerce participants into treatment. Participants will also have free access to the self-help tool for 6 months from the time they started using e-couch.

As the study does not exceed minimal risk and given the availability and safety of the self-help tool we will use, we will not provide or pay for ancillary care during or after the trial beyond what has been described above.
