# Supplementary material for: Protocol for a randomized trial to predict the efficacy of cognitive and behavioral interventions for symptoms of depression
Source: Front Psychiatry. 2026 Apr 16;17:1774560. doi: 10.3389/fpsyt.2026.1774560 (PMC13128560; doi:10.3389/fpsyt.2026.1774560)
Supplement: Supplementary file 1 [file DataSheet1.pdf]

Supplement to ‘Protocol for a Randomized Trial to Predict the Efficacy of Cognitive and Behavioral Interventions for Symptoms of Depression’

Supplementary methods

0.1 Protocol changes

Protocol version 1.0 differed from the current version in terms of eligibility criteria and the number of tasks and questionnaires.

In protocol version 1.0, we used the following inclusion criteria:

- at least 18 years old,
- physically located in the US,
- fluent in English,
- not be receiving psychotherapy or plan to begin one within the next eight weeks,
- had not changed the doses or type of antidepressants in the past four weeks, or plan to make such changes in the next eight weeks, and
- their primary mental health concern was to improve their mood, reduce negative thoughts, enjoy more activities again, or reduce symptoms of depression.

We did not exclude participants who failed the attention checks at the beginning of the study, nor did we conduct identity verification over Zoom. The first 100 participants were all assigned to the training dataset, such that we could conduct an initial data quality check. After we examined the data quality, we updated the inclusion and exclusion criteria, and incorporated the identity checks. We also added criteria to ensure that no more than one individual from the same household is enrolled in the current study.

Some tasks and questionnaires that are now in version 1.1 were not included in version 1.0, including the symmetry span task, NEO-FFI, PSWQ, MSPSS, and the study motivation question. In version 1.0, participants repeated the Matrix reasoning task with identical stimuli.

Adaptations of standardized questionnaires

We made the following adaptations to standardized questionnaires:

- QIDS: Item 12 from the original QIDS-SR 16, which asks about suicidality, was removed.
- NEO-FFI: We only used item 1, 6, 11, 26, 36, 41, 51 from the original scale.
- SHAPS: Minor edits were made to questions 1 and 8 to adjust unfamiliar British connotations of words for an American sample or to modernize.
- WHO-5: We updated the original instruction with more explanation as follows :“Please indicate for each of the 5 statements which is closest to how you have been feeling over the past 2 weeks. Notice that higher numbers mean better well-being. Example: If you have felt cheerful and in good spirits more than half of the time during the last two weeks, select the answer 3.” We also made slight modifications to the text on the scale.

- WSAS: We modified the text on the number scale (i.e., 0 (Not at all), 2 (Slightly), 4 (Definitely), 6 (Markedly), and 8 (Very severely), with the exception of item 1 where 8 indicated very severely impaired to the point I can not work.
- RTQ: We made minor edits to items 4-6 to reflect the context of e-couch rather than therapist-led treatment (e.g. changing 'therapy' to 'self-help course'; 'session' to 'module').

Study-specific measures

We developed the eligibility questionnaire, the pain questionnaire, the return of symptoms questionnaire, the psychiatric treatment questionnaire, the study motivation questionnaire, and the past depression questionnaire (all listed below) to capture information relevant for our study for which not standardized questionnaires exist.

Eligibility questionnaire

Please note that we also include healthy volunteers into the study. You do not have to have any symptoms to pass this screening. It is very important that you answer all questions honestly.

- How old are you?
- Postal / zip code in which you are physically based (free text entry)
- Country in which you are physically based (Option: USA, Canada, Australia, Other)
- Are you fluent in English?
- Do you have a severe hearing problem?
- Do you have headphones that you can use for an experiment within the next week?
- Have you previously participated in a study from the Niv Lab or the Princeton Study (Behavioral study to predict the efficacy of a self-help tool) before?
- Have you previously completed this screening survey before?
- Has anyone in your household participated, or is still participating in this study?
- Do you share a computer with someone who is participating in this study?
- Do you have a picture ID and would be able to have a brief video zoom meeting with us to verify your identity, if selected for such an identity check?
- How did you learn about this study? (Option: Facebook, Twitter, Instagram, Reddit, TikTok, ResearchMatch, Website of Nivlab, Recommended by a friend, USA Today - print, USA Today - online, Cint, Other)
- If you choose other in the last question, please specify. (free text entry)
- Are you currently undergoing psychotherapy or do you regularly engage with internet-delivered psychotherapy interventions such as a self-help app to improve your mental health? [Yes/No]
- Do you have an appointment to start psychotherapy in the next 8 weeks, or are you planning to make one? [Yes/No]
- Are you currently taking antidepressants? [Yes/No]

If you choose Yes in the last question,

- Have you changed the doses or type of antidepressant you are taking within the last four weeks? [Yes/No]
- Are you planning to change your antidepressant medication within the next 8 weeks? [Yes/No]

What is your main mental health concern that you want to work on?

- I would like to improve my mood.
- I would like to reduce negative thoughts.
- I would like to enjoy more activities again.

- I would like to reduce symptoms of depression.
- I would like to be less anxious/afraid.
- I would like to think/be reminded less of traumatic events.
- I would like to improve my sleep.
- I would like to reduce the amount of alcohol/substances I am consuming.
- I would like to be less afraid/feel more confident around others.
- I would like to reduce chronic pain.
- Other
- I have no mental health concerns.

Pain Questionnaire

1. Did you experience any pain within the last week?
    - If yes, where in your body did you experience the most intense pain?
2. Did you experience other pain last week that started a longer time ago (e.g., weeks, months, or years ago)?
    - If yes, where in your body did you experience pain for the longest time?
3. For one or two locations of pain (depending on the answers above), please rate:
    - Pain intensity (VAS scale)
    - Pain unpleasantness (VAS scale)
    - Pain interference (VAS scale)
4. How long have you been in pain?
    - I am not in pain
    - <2 weeks
    - 2–4 weeks
    - 1–3 months
    - 3–6 months
    - 6–12 months
    - 1–5 years
    - Over 5 years
    - Over 10 years
5. Did your pain start with an injury, surgery, infection, or childbirth?
    - Yes, an injury
    - Yes, a surgery
    - Yes, an infection
    - Yes, childbirth
    - Multiple
    - No concrete pain start
    - None of the above

Return of symptoms questionnaire

We would like you to reflect on your well-being and mood over two time periods: 1) how you felt at the start of the study; 2) a recent two-week period from between x (variable from 2 weeks to 6 months, depending on the study stage) until now, where you have felt the least well with regard to your mood, happiness, and/or enjoyment and interest in doing fun activities. In comparison, was your well-being and mood in this recent two-week period

- Better than when you started the study

- Similar to when you started the study
- Worse than when you started the study
- Not well, but different than when you started the study

Psychiatric treatment questionnaire

This questionnaire will ask you about your treatment. Please **do not** provide any form of personal information or information that may identify you (e.g. name of the treatment provider, your medical record number).

Section 1: Diagnoses

1. Have you ever been diagnosed with a mental health condition? [Yes/No]
2. If you choose [Yes] to the last question, would you be willing to share your mental health diagnosis with us? [Yes/No]
3. If you choose [Yes] to the last question, have you ever been diagnosed with any of the mental health conditions below? Please select all that apply (including past and/or current diagnoses), or leave blank if you prefer not to say.

- Adjustment disorder
- Agoraphobia
- Alcohol dependence
- Anorexia nervosa
- Attention deficit hyperactivity disorder (ADHD)
- Autism spectrum disorder
- Binge eating disorder
- Bipolar I disorder
- Bipolar II disorder
- Body dysmorphic disorder
- Borderline personality disorder
- Bulimia nervosa
- Dysthymia
- Eating disorder (not otherwise specified)
- Generalized anxiety disorder (anxiety)
- Major depressive disorder (depression)
- Obsessive-compulsive disorder (OCD)
- Panic disorder
- Other personality disorders
- Post-traumatic stress disorder (PTSD) (trauma)
- Premenstrual dysphoric disorder
- Psychotic disorder
- Schizoaffective disorder
- Schizophrenia
- Schizotypal personality disorder
- Social anxiety disorder
- Somatic symptom disorder
- Specific phobia
- Substance dependence / addiction
- Tourette syndrome
- Trichotillomania

- Other diagnosis (Free text entry; Please do **not** include personal or identifiable information)

Section 2.1: Past Antidepressant Use

Have you taken any antidepressant medications in the last *x* (variable from 2 weeks to 12 months, depending on the study stage)? [Yes/No]

If yes, please select all medications taken within the last four months:

- Anafranil (clomipramine)
- Asendin (amoxapine)
- Aventyl (nortriptyline)
- Celexa (citalopram hydrobromide)
- Cymbalta (duloxetine)
- Desyrel (trazodone HCl)
- Elavil (amitriptyline)
- Effexor (venlafaxine HCl)
- Emsam (selegiline)
- Etrafon (perphenazine/amitriptyline)
- Fluvoxamine maleate
- Lexapro (escitalopram hydrobromide)
- Limbitrol (chlordiazepoxide/amitriptyline)
- Ludiomil (maprotiline)
- Marplan (isocarboxazid)
- Nardil (phenelzine sulfate)
- Nefazodone HCl
- Norpramin (desipramine HCl)
- Pamelor (nortriptyline)
- Parnate (tranylcypromine sulfate)
- Paxil (paroxetine HCl)
- Pexeva (paroxetine mesylate)
- Prozac (fluoxetine HCl)
- Remeron (mirtazapine)
- Sarafem (fluoxetine HCl)
- Seroquel (quetiapine)
- Sinequan (doxepin)
- Surmontil (trimipramine)
- Symbyax (olanzapine/fluoxetine)
- Tofranil (imipramine)
- Tofranil-PM (imipramine pamoate)
- Triavil (perphenazine/amitriptyline)
- Vivactil (protriptyline)
- Wellbutrin (bupropion HCl)
- Zoloft (sertraline HCl)
- Zyban (bupropion HCl)
- Other antidepressant (free text) (please do **not** include personal or identifiable information)

For each antidepressant medication, please specify:

1. Did you receive the treatment for a specific diagnosis? [Yes/No]
   If yes, please select all that apply (see list of diagnoses above).

2. Start date [calendar date]
3. End date (leave blank if not applicable) [calendar date]
4. Dosage per time point [Format: numerical field for dosage + unit selection]:
   - Morning:
   - Midday:
   - Evening:
   - Night:
5. Did you receive any additional instructions on how to take the medication? (free text)
6. How often did you not take your medication within the last four weeks?
   - Not at all
   - Several days
   - Over half the days
   - Nearly every day

Section 2.2: Future Antidepressant Use

Do you plan to take any antidepressant medications in the next *y* (variable from 12 months to 1 week, depending on the study stage; *x* in 2.1 and *y* adds up to around 12 months which is the length of the study)? [Yes/No]

If yes:

1. Do you intend to receive treatment for a specific diagnosis? [Yes/No]
2. If yes, please select all that apply (see list of diagnoses above)
3. Intended start date [calendar date]

Section 3.1: Past Psychotherapy

Have you received any psychological therapy/psychotherapy treatment within the last *x* (variable from 2 weeks to 12 months, depending on the study stage)? [Yes/No]

If yes, please select all therapy types received within the last four months:

- Cognitive-behavioral therapy
- Cognitive-Behavioral Analysis System of Psychotherapy
- Behavioral Activation
- Cognitive Restructuring
- Exposure therapy
- Psychodynamic therapy
- Psychoanalytical therapy
- Acceptance and Commitment therapy
- Body psychotherapy
- Emotionally focused therapy
- Existential therapy
- Family therapy
- Hypnotherapy
- Interpersonal psychotherapy
- Mindfulness-based cognitive therapy
- Metacognitive therapy
- Systemic therapy
- Trauma-focused therapy
- Eye movement desensitization and reprocessing (EMDR)

- Narrative exposure therapy
- Other therapy (free text) (please do **not** include personal or identifiable information)

For each psychotherapy, please specify:

1. Did you receive treatment for a specific diagnosis? [Yes/No]
   If yes, select all that apply (see list of diagnoses above)
2. Start date [calendar date]
3. End date (leave blank if not applicable) [calendar date]
4. Please describe what you do in your therapy in general terms (free text)
5. Setting:
   - Individual in-person therapy
   - Group in-person therapy
   - Individual therapy via video-conference (e.g., Zoom)
   - Text-based therapy via e-chat, email, or text
   - Online modules without therapist interaction
   - Other setting (please do **not** include personal or identifiable information)
6. How often did you attend therapy sessions since you started this therapy on average? [numeric field for number+ unit (e.g., per week)]
7. How often did you not attend a recommend/scheduled sessions in the last four weeks?
   - Never
   - A few sessions
   - More than half
   - Almost all

Section 3.2: Future Psychotherapy

Do you plan to receive psychological therapy within the next y (variable from 12 months to 1 week , depending on the stage of the study)? [Yes/No]

If yes:

1. Do you plan to receive treatment for a specific diagnosis? [Yes/No]
2. If yes, select all that apply (see list of diagnoses above)

Section 4: General Questions

1. What is your weight? [numeric + unit (kg/lb)]
2. Do you get any other treatment for mental health? (please do **not** include personal or identifiable information) (free text)

Study motivation questionnaire

Why are you interested in participating in our study? Please indicate on the slider from 0-100 to which extent the stated reason is why you want to participate in the study (the following options are presented in different pages, participants can not go back to the previous page):

- I want to improve my mood.
- I want to earn money.
- I want to contribute to research.
- I want to improve my well-being.

Please describe why you are motivated to participate in this study and what you hope to gain from your participation (free text). (Please do not include any personal information.)

Past depression questionnaire

Please answer the following questions.

- How old were you when you felt depressed for more than two weeks for the first time in your life?
- How many months have you been feeling depressed in your entire life (regardless of when or if you received a diagnosis of a depressive disorder)?
- If you are currently feeling depressed, how many months has this episode lasted (regardless of when or if you received a diagnosis of a depressive disorder)?

Mental health resources page

At every instance where participants report mental health symptoms or are exposed to emotional stimuli, information about available mental health resources will be presented to them as shown in Fig. 1.

Thank you for sharing your responses to the questionnaires with us. As a reminder, these responses will remain strictly anonymous; indeed your identity is not known to the experimenters.

If you are interested in learning more about mental health in general or in speaking to a professional, we have provided some resources below.

- To learn more about mental health conditions, treatment, research, and warning signs, you can visit the NAMI site https://www.nami.org/Learn-More
- If the questions you answered trouble you in any way, we urge you to arrange a timely appointment with your physician, clinical psychologist or psychiatrist.
- For help finding a mental health professional, you can visit https://www.nami.org/Find-Support/Living-with-a-Mental-Health-Condition/Finding-a-Mental-Health-Professional, and/or email info@nami.org for more specific or personal concerns.
- A number of organizations offer alternative sources of support through difficult times and/or challenges. Below we have listed some examples of these free and confidential support networks:
    - The Lifeline Crisis chat (https://suicidepreventionlifeline.org/chat).
    - The National Suicide Prevention Hotline (http://suicidepreventionlifeline.org, call:1-800-273-8255)

You may wish to print this screen for your reference.

Figure 1: Mental health resources provided to participants post-questionnaires and post-experiment.
